# Estrus Prediction Models for Dairy Gyr Heifers

**DOI:** 10.3390/ani11113103

**Published:** 2021-10-30

**Authors:** Valesca Vilela Andrade, Priscila Arrigucci Bernardes, Rogério Ribeiro Vicentini, André Penido Oliveira, Renata Veroneze, Aska Ujita, João Alberto Negrão, Lenira El Faro

**Affiliations:** 1 Beef Cattle Research Center, Institute of Animal Science (IZ), Rod Carlos Tonani, Km 92, Sertãozinho 14174-000, Brazil; valescavilela@hotmail.com (V.V.A.); priscilaarrigucci@gmail.com (P.A.B.); rog.vicentini@hotmail.com (R.R.V.); aska_ujita@hotmail.com (A.U.); 2 Agricultural Research Company of Minas Gerais (EPAMIG), Rua Afonso Rato 1301, Uberaba 38001-970, Brazil; andre.penido@gmail.com (A.P.O.); veronezerenata@gmail.com (R.V.); 3 Faculty of Animal Science and Food Engineering (FZEA), University of São Paulo (USP), Av. Duque de Caxias Norte 225, Pirassununga 13635-900, Brazil; jnegrao@usp.br

**Keywords:** activity, body temperature, heat, reproduction, sensors, Zebu

## Abstract

**Simple Summary:**

Intraruminal devices are already being used to predict reproductive events in cattle. For this prediction, several models and approaches can be used. The aim of this study was to evaluate changes in rumen reticulum temperature (RRT) and activity (ACT) during estrus in Dairy Gyr heifers and to evaluate different models for estrus prediction. There was an increase in both RRT and ACT in the estrus period compared to the same period on the day before and the day after estrus. Among the mathematical models, Random Forest had the best performance. The present results suggest that RRT and ACT can contribute to the identification of estrus and be of value for improving the reproductive efficiency of Zebu herds in tropical regions.

**Abstract:**

Technological devices are increasingly present in livestock activities, such as identifying the reproductive status of cows. For this, predictive models must be accurate and usable in the productive context. The aims of this study were to evaluate estrus-associated changes in reticulo-rumen temperature (RRT) and activity (ACT) in Dairy Gyr heifers provided by reticulo-rumen boluses and to test the ability of different models for estrus prediction. The RRT and ACT of 45 heifers submitted to estrus synchronization were recorded using reticulo-rumen boluses. The means of RRT and ACT at different time intervals were compared between the day before and the day of estrus manifestation. An analysis of variance of RRT and ACT was performed using mixed models. A second approach employed logistic regression, random forest, and linear discriminant analysis models using RRT, ACT, time of day, and the temperature-humidity index (THI) as predictors. There was an increase in RRT and ACT at estrus (*p* < 0.05) compared to the same period on the day before and on the day after estrus. The random forest model provided the best performance values with a sensitivity of 51.69% and specificity of 93.1%. The present results suggest that RRT and ACT contribute to the identification of estrus in Dairy Gyr heifers.

## 1. Introduction

Estrus detection in cattle is important for the success of dairy herds that use artificial insemination [1]. However, the accuracy of estrus detection is low in Zebu breeds [2] since the duration of estrus is shorter when compared to European breeds [3,4]. According to Wiltbank et al. [5] and Layek et al. [6], estrus manifestation with weak signals, of short duration, and of low intensity has been reported in Zebu breeds, attributed to low circulating levels of 17β- estradiol. In addition, Pinheiro et al. [7] observed that, in Dairy Gyr cows, estrus mainly occurs between the beginning of the night and early morning hours, between 6 pm and 6 am. Thus, the visual detection of estrus is difficult because it is labor intensive and time consuming mainly in extensive cattle production systems.

The adoption of monitoring technologies for estrus detection could be an alternative to improve the reproductive efficiency of herds. Some studies have suggested the use of body temperature as a predictor of estrus and calving in cattle. These studies demonstrated an increase of up to 0.4 °C in different regions of the animal’s body (e.g., rectal, vaginal, epithelial, or reticulo-rumen) during estrus and a drop of up to 1 °C during calving [8,9,10,11]. Within this context, among the different existing precision technologies to automate and facilitate the detection of estrus, minimizing the use of labor, internal temperature sensors placed in the rumen of the animals have been intensively studied [9,12,13,14].

The use of these technologies for the collection and storage of information generates a large amount of data. Computational tools are used for the interpretation and analysis of these data. Among the most widely used tools, machine learning is a good candidate for data mining and for the development of prediction models [15]. The predictive potential of this technique is evaluated by a comparison of the events identified by the algorithm of the technology with a gold standard, for example, visual observation of estrus, measurement of progesterone levels in blood or milk, ultrasonography, or a combination of these methods [1]. This study addresses the hypothesis that reticulo-rumen temperature and physical activity changes during estrus and can be used as predictive variables in prediction models.

Within this context, the aims of the present study were to evaluate estrus-associated changes in internal body temperature and activity in Dairy Gyr heifers using continuous data provided by reticulo-rumen boluses, and to test the ability of different models to predict the occurrence of estrus.

## 2. Materials and Methods

The experiment was approved by the Ethics Committee (CEU) of Instituto de Zootecnia (protocol number 230-16) and was carried out at the Campo Experimental Getúlio Vargas, Empresa de Pesquisa Agropecuária de Minas Gerais (EPAMIG), located in Uberaba, Brazil (19°44′54″ S latitude, 47°55′55″ W longitude, and altitude of 801 m). The experimental period comprised the months of October and November 2016. The climate data including minimum and maximum environmental temperatures and relative air humidity were collected at the meteorological station of Instituto Nacional de Meteorologia (INMET), near the experimental area. The temperature-humidity index (THI) used in the present study was calculated as proposed by Mader et al. [16] and described by Vicentini et al. [17], as this environmental parameter can influence reticulo-rumen temperature. 

### 2.1. Animals

**Figure 1:**
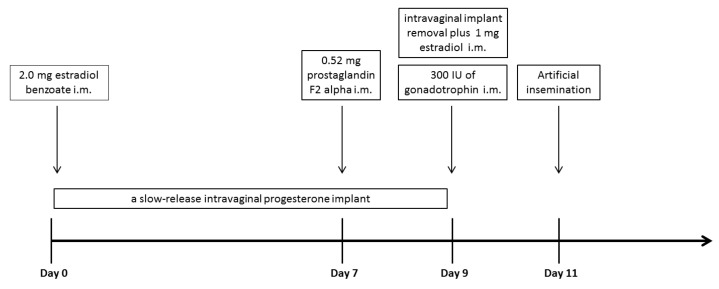
Hormonal protocol for estrus synchronization and artificial insemination. i.m: intramuscular administration.

Forty-five nulliparous Dairy Gyr females (*Bos taurus indicus*) with a mean age of 3.4 + 0.5 years, ranging from 30 to 56 months, were used in this study. The animals were managed on the *Urochloa decumbens* pasture, with water and mineral salt ad libitum. The heifers were submitted to an 11-day hormonal protocol for estrus synchronization, followed by artificial insemination (Figure 1). To facilitate the visualization of estrus behavior in the observation paddock, the animals were divided into four synchronization groups. Thus, there was an interval of 7 days in the application of the protocols between groups. The following protocol was used in the present study: day 0: 2.0 mg estradiol benzoate (Synchrodiol, Ouro Fino, São Paulo, Brazil) intramuscularly (i.m.) plus a slow-release intravaginal progesterone implant (1 g; Sincrogest, Ouro Fino, São Paulo, Brazil); day 7: 0.52 mg prostaglandin F2 alpha (sodium cloprostenol; Sincrocio, Ouro Fino, São Paulo, Brazil) i.m.; day 9: intravaginal implant removal plus 1 mg estradiol (SincroCp, Ouro Fino, São Paulo, Brazil) and 300 IU of gonadotrophin (eCG Syncro, Ouro Fino, São Paulo, Brazil) i.m. Artificial insemination was performed on day 11. Further information can be obtained from Vicentini et al. [17].

### 2.2. Sexual Behavior

The main behavior sign of estrus in Zebu cows is the acceptance of mounts, which is defined as standing immobile to be mounted by another animal, indicating sexual receptivity [18]. Following the method of Pires et al. [19], the first mount acceptance (standing heat) was defined as the first mount within an interval of less than 90 min of a sequence of mounts. The time when the first mount acceptance was observed was defined as the onset of estrus in each animal. Thus, the sexual behavior of females was recorded visually every 10 min by a group of eight trained observers, in six-hour shifts with two people at each time, over a period of 36 h for the determination of estrus onset [17]. Observations started 12 h after the removal of the progesterone implant (day 9) and finished at 48 h on AI (day 11). For a better visualization of sexual behavior, the animals remained in a paddock containing an area of 0.45 ha, close to the management corral.

Transrectal ultrasonography (Siui CTS 900v, Shantou, China) was performed every 12 h, starting 48 h after the removal of the progesterone implant and terminating 60 h after implant removal. Thus, ovulation of the animals was confirmed by ultrasound based on the absence of the dominant follicle, 60 h after the progesterone device removal. All females used in the present study ovulated and were sexually receptive, characterizing the occurrence of estrus.

### 2.3. Body Temperature and Activity Recording

Temperature- and activity-sensing boluses (TX-1442, Smaxtec Animal Care, Graz, Austria) were placed in the reticulo-rumen of each female using a custom balling gun 45 days before estrus synchronization, as described by Vicentini et al. [20]. The bolus device measured 105 mm × 35 mm, weighed 0.21 kg, and was ruminal-fluid resistant. The reticulo-rumen temperature (RRT) and accelerometer-based activity (ACT) of the animals were continuously recorded every 10 min. The data were collected and transmitted to online servers by a telemetry system through an antenna with a range of 30 m. The readings performed by the antenna were accessed using the Smaxtec Messenger software. 

### 2.4. Statistical Analysis

Statistical analyses were performed to identify changes in mean RRT at different time intervals before and after the first mount acceptance; the analyses were thus divided into two schemes. The time when the animal showed the first sexual receptivity was called hour 0 RRT. Different daily hours before first mount acceptance were then used to define Scheme 1 and different daily hours after acceptance to define Scheme 2. 

**Figure 2:**
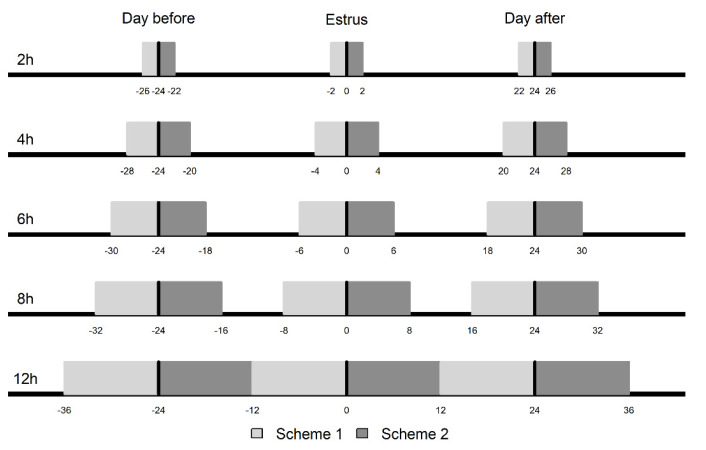
Time intervals (hour) during which mean reticulo-rumen temperatures were compared in relation to first observed mount acceptance (0). Scheme 1 (light gray)—2 h: Interval of 2 h before first observed mount acceptance was compared with the same daily hours the day before (−26 to −24 h) and the day after (22 to 24 h). 4 h: Interval of 4 h before first observed mount acceptance was compared with the same daily hours the day before (−28 to −24 h) and the day after (20 to 24 h). 6 h: Interval of 6 h before first observed mount acceptance was compared with the same daily hours the day before (−30 to −24 h) and the day after (18 to 24 h). 8 h: Interval of 8 h before first observed mount acceptance was compared with the same daily hours the day before (−32 to −24 h) and the day after (16 to 24 h). 12 h: Interval of 12 h before first observed mount acceptance was compared with the same daily hours the day before (−36 to −24 h) and the day after (12 to 24 h). Scheme 2 (dark gray)—2 h: Interval of 2 h after first observed mount acceptance was compared with the same daily hours the day before (−24 to −22 h) and the day after (24 to 26 h). 4 h: Interval of 4 h after first observed mount acceptance was compared with the same daily hours the day before (−24 to −20 h) and the day after (24 to 28 h). 6 h: Interval of 6 h after first observed mount acceptance was compared with the same daily hours the day before (−24 to −18 h) and the day after (24 to 30 h). 8 h: Interval of 8 h after first observed mount acceptance was compared with the same daily hours the day before (−24 to −16 h) and the day after (24 to 32 h). 12 h: Interval of 12 h after first observed mount acceptance was compared with the same daily hours the day before (−24 to −12 h) and the day after (24 to 36 h).

In Scheme 1 (Figure 2), the mean RRT values at 2, 4, 6, 8, and 12 h before the first observed mount acceptance (hour 0 RRT) were statistically compared to the mean values obtained on the same daily hours for the days before and after estrus. 

Scheme 2 (Figure 2) considered the same intervals (2, 4, 6, 8, and 12 h) and periods (day before, estrus, and day after) as Scheme 1; however, the analyses consisted of statistically comparing the mean RRT values at 2, 4, 6, 8, and 12 h after the first observed mount acceptance (hour 0 RRT) with the mean values obtained on the same daily hours for the days before and after estrus.

The mean RRT value per hour was calculated for each animal and included in the model as the dependent variable. For each scheme (1 = before; 2 = after first observed mount acceptance), the model included the fixed effects of classes of period (day before, estrus, and day after), time (each hour within the intervals of 2, 4, 6, 8, and 12 h), period × time interaction, and protocol group to which the animals belonged (four groups). In addition, the THI per hour and time of day (0 to 23:00 h) were included as covariates (linear effects). RRT values < 37.7 °C were associated with water consumption and were excluded from the analyses [9,21].

**Figure 3:**
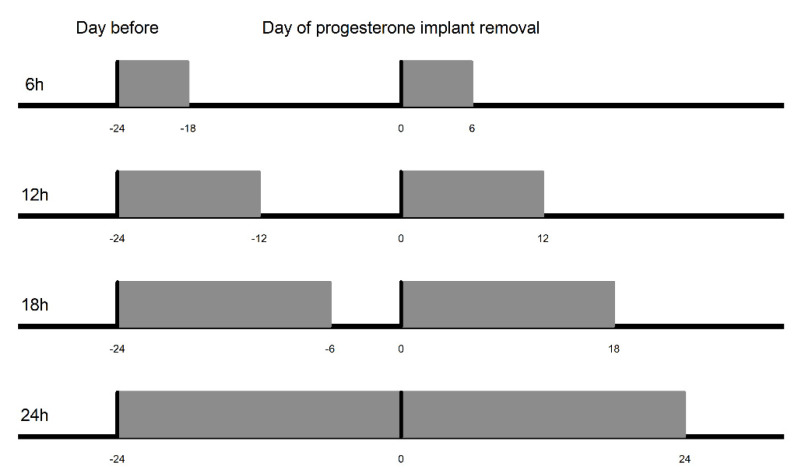
Time intervals (hour) during which mean activity per hour was compared in relation to removal of the progesterone implant (0). 6 h: Interval of 6 h after implant removal was compared with the same hours of the day before (−24 to −30 h). 12 h: Interval of 12 h after implant removal was compared with the same hours of the day before (−24 to −36 h). 18 h: Interval of 18 h after implant removal was compared with the same hours of the day before (−24 to −42 h). 24 h: Interval of 24 h after implant removal was compared with the same hours of the day before (−24 to −48 h).

To detect changes in the pattern of the mean ACT (Figure 2) before estrus manifestation, different daily hours compared during different periods were also considered in the statistical analyses. The time of removal of the progesterone implant in each animal was defined as hour 0 ACT (reference point) and was considered a benchmark to check if there were differences in activity that preceded the first signs of estrus. The time intervals considered for the analysis of changes in ACT were: up to 6, 12, 18, and 24 h after implant removal (hour 0 ACT). Thus, the mean ACT during these intervals on the day of the implant removal was compared statistically with the mean ACT on the same daily hours the day before the implant removal (Figure 3). Records that could cause changes in the activity of animals due to handling and therefore not related to the occurrence of estrus were eliminated from datasets. The classificatory effects of period (day before and day of implant removal), time (each hour within the interval of 6, 12, 18, and 24 h), period × time interaction, and protocol group to which the animals belonged (four groups) were included in the model. The effects of THI (per hour) and time of day (0 to 23:00 h) were included as covariates (linear effect).

Normality and homoscedasticity tests were carried out for both variables, RRT and ACT. However, ACT was transformed on a base 10 logarithmic scale (log10) due to the lack of normality of this variable and residuals. Analyses of variance of RRT and ACT were performed with mixed linear models to evaluate repeated measures using the lme function of the R software (R Development Core Team, 2008). 

The least square means estimated for the classificatory effects of period and time were compared by the Tukey–Kramer test at a significance level of 5% using the emmeans package of the R software (R Development Core Team, 2008).

For the analysis of RRT and ACT, the residuals were modeled considering the covariance between repeated measures of the same animal at the time intervals by employing different residual (co)variance structures (corAR1, corARMA, corCAR1, corCompSymm, corExp, corGaus, corLin, corRatio, corSpher, corSymm), and the best structures were chosen based on Akaike’s information criterion (AIC).

### 2.5. Prediction Study

Logistic regression, random forest, and linear discriminant analysis models were compared for the prediction of estrus occurrence. The response variables were assumed to have a binary distribution, with Yi = 1 for the presence of estrus or Yi = 0 for the absence of estrus. The effects of RRT and ACT obtained every 10 min, time of day, and the THI were included in the model as independent variables. The mean and standard deviation of the THI on the days considered for this study and for the statistical analysis are described in Table 1.

The period considered for prediction was determined in such a way as to allow the simulation of a real situation of sensor monitoring for the identification of estrus in the animals; thus, this period was not restricted only to the time close to the manifestation of sexual receptivity. For this purpose, data comprising the period between implant removal, which occurred at 20:00 h on day 9, and 00:00 h of the day following insemination, which occurred on day 11 of the ovulation synchronization protocol, were used, totaling 52 h. The response variable presence of estrus (Yi = 1) was considered based on behavioral observations from the first to the last accepted amount; responses in a different period were defined as the absence of estrus (Yi = 0). The dataset contained on average of 45.11 records of the presence of estrus (Yi = 1) and 263.38 records of the absence of estrus (Yi = 0) per animal. The onset of estrus manifestation occurred on day 11 of the synchronization protocol, with estrus manifestation starting during the day in 21 animals and during the night in 24 animals. 

Cross-validation as described by Borchers et al. [22] was used for the analysis of all models. For this purpose, the 45 animals were randomly allocated into five-fold cross-validation, with 9 animals in each group. Thus, the effects of the predictors were estimated in four groups (80% of the observations), defined as the training set, and the predictive ability of each model was tested in one validation group (20% of the observations). The analyses were repeated five times; in each repetition, a different group was considered the test set, and the other groups were used as the training set. The analyses were performed five times for each model; thus, each subset was considered as validation group once. 

Then, the values observed for the response variable (presence or absence of estrus) and the values predicted by the tested model were used to calculate the confusion matrix. This matrix supported the evaluation of the models’ performance by the metrics as described by Borchers et al. [22]: sensitivity, specificity, the positive predictive value (PPV), and the negative predictive value (NPV), as well as accuracy and the AUC. The last parameter corresponds to the area under the ROC curve whose maximum value is 1.0, indicating a perfect test, i.e., 100% sensitive and 100% specific [23]. The AUC was obtained using the ROCR function of the R program (R Development Core Team, 2008). These metrics are reported as the mean repeat number of the cross-validation analyses, which were performed five times.

## 3. Results

### 3.1. Daily Variation of Reticulo-Rumen Temperature (RRT) and Activity (ACT)

**Figure 4:**
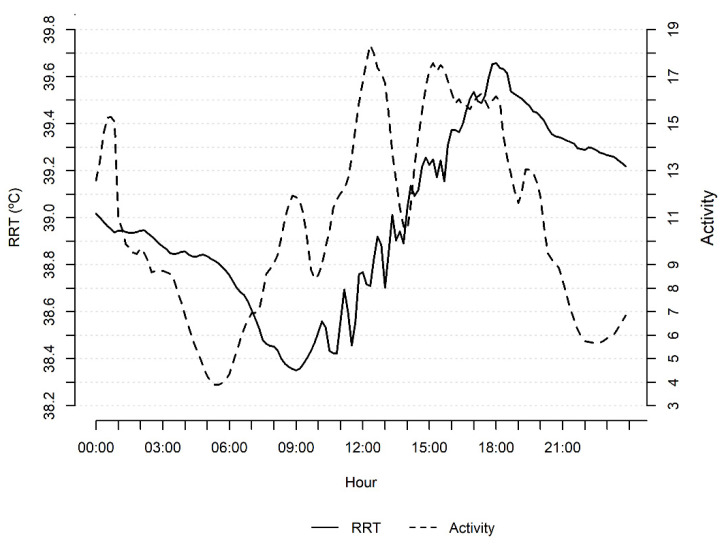
Daily mean of reticulo-rumen temperature (RRT) and activity of Zebu heifers on a day.

Daily means of RRT and ACT have daily variation (Figure 4) according to the circadian rhythm, indicating the importance of including time of day in prediction models. Changes in RRT were observed, with the body temperature increasing during the day, reaching a peak at the end of the day, and declining during the night. Changes in ACT were more frequent during the day, and the peak of activity was observed between 12:00 to 18:00 h, shortly before the observed peak for RRT, probably related to grazing activities. 

### 3.2. Estimated Means and Standard Errors for Reticulo-Rumen Temperature (RRT) and Activity (ACT)

Comparing all time points in Scheme 1 (hours before estrus), the estimated mean RRT values during estrus were similar to RRT on the same daily hours the day before (*p* > 0.05) and differed from those at 2, 4, 6, and 8 h (*p* < 0.05) on the day after (Table 2). An analysis of variance in Scheme 2 (hours after estrus) showed higher (*p* < 0.05) estimated mean RRT vales during estrus for all times studied when compared to the mean values on the day before and after estrus (Table 2).

**Figure 5:**
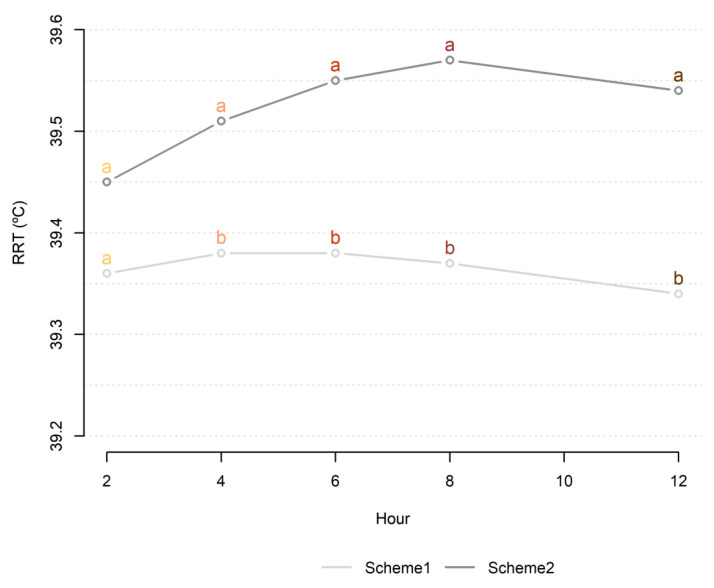
Estimated least squares means of RRT by hour during estrus in Scheme 1 (hours before) and Scheme 2 (hours after). Means in the same color followed by different superscript letters differ significantly (Tukey test, 5% significance level).

Analyzing the estrus period (Table 2), the mean RTT values at the times before hour 0 RTT (first observed mount acceptance) were generally lower (39.34 to 39.38 °C) than those observed at the times after 0 RRT (39.45 to 39.57 °C). These differences suggest that the increase in RTT occurs after the first mount acceptance, especially between 6 and 12 h, peaking at 8 h after 0 RRT (Figure 5).

**Figure 6:**
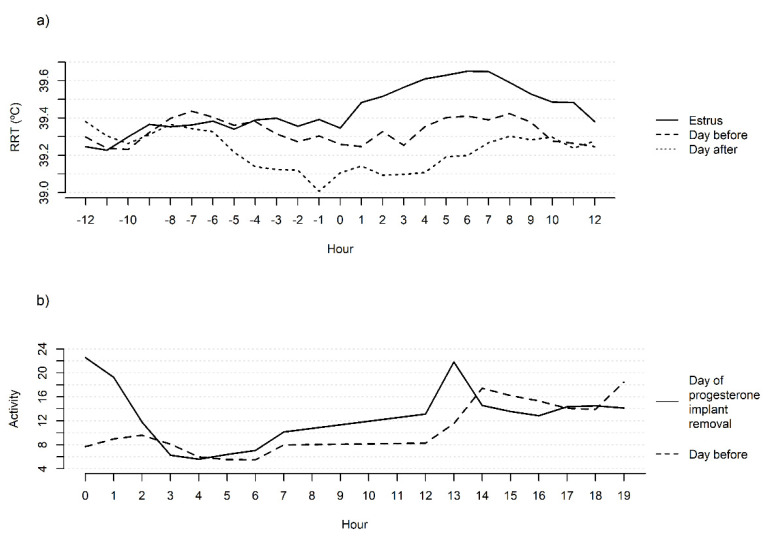
(**a**) Observed mean reticulo-rumen temperatures (RRT) over the hours before and after first observed mount acceptance (0) on the day of estrus, on the day before, and on the day after. (**b**) Observed mean activity over the hours after removal of the progesterone implant (hour 0) and on the day before.

The estimated mean ACT values were higher (*p* < 0.05) at all time points (6, 12, 18, and 24 h) after the implant removal when compared to the same periods the day before (Table 3), with an average difference of 2.17 units. Figure 6b graphically illustrates these differences in the increase in ACT after the implant removal (hour 0). The increase in ACT during this period can be attributed to the behavioral changes that characterize and precede the primary sign of estrus, such as the acceptance of mounts.

### 3.3. Prediction Study

Among the three tested models, logistic regression and linear discriminant analyses showed poor performance in predicting estrus events, as indicated by the metrics used to evaluate the performance of the prediction models (Table 4). However, the random forest model was able to predict the events that occurred and showed the best predictive ability of estrus occurrence (Table 4).

The sensitivity of the random forest model was 51.69%, and the PPV was 56.02%, while these metrics were close to zero for the other models (Table 4). Although the models studied had greater difficulty in predicting estrus events, they were good predictors of non-estrus, with specificities of 99.79%, 93.10%, and 99.65% for the logistic regression, random forest, and linear discriminant analysis, respectively (Table 4). These percentages can be attributed to the observation period in the present study, totaling 52 h. 

The accuracy of estrus prediction of the tested models was 85%, 87%, and 85% for the logistic regression, random forest, and linear discriminant analysis, respectively (Table 4). In addition to the metrics derived from the confusion matrix and accuracy, the AUC was also used to assess the performance of the tested models (Table 4). Using this measure, the best performance (AUC = 0.90) was obtained for the random forest model. The maximum value for the AUC is 1.0, which indicates a perfect test, i.e., 100% sensitive and 100% specific [23].

## 4. Discussion

The elevation of RRT may be attributed to the occurrence of estrus, i.e., the period when females are sexually receptive. In fact, according to Sellier et al. [24], the variations in animal reproduction accompany the animal’s body temperature. Although rectal temperature is the most common measure of central body temperature, the high and significant correlation (r = 0.92) between intraruminal and rectal temperatures [25] suggests similar temperatures in these regions. The results of RRT analyses suggest that devices inserted into the reticulo-rumen of cattle can be used as auxiliary tools for detecting estrus in the animals.

The differences in mean RRT values on the day before and after estrus (Figure 6a) were on average 0.22 °C, while the differences between the day of estrus and the day after were 0.36 °C. In beef heifers, Randi et al. [26] observed a significant increase of 0.4 ± 0.1 °C in body temperature during estrus, and Dolecheck et al. [1] found that the RRT measured by bolus sensors increased 0.43 °C during estrus in lactating cows. Both values obtained for taurine breeds are higher than those obtained in the present study. Using a larger dataset but including data from the present study, Vicentini et al. [17] attributed the increase in RRT and rectal and vaginal temperatures to the occurrence of estrus. 

Estrus is a period that comprises a set of physiological and behavioral signals [27] and is triggered by an increase in estradiol and a decrease in progesterone [28]. These hormonal alterations were observed by some authors such as Wettemann et al. [29] and Clapper et al. [30] who reported changes in the levels of the luteinizing hormone and estradiol before, during, and after estrus and also variations in body temperature during this period [9,30]. In addition to the possible relationship between the luteinizing hormone peak and temperature variations also reported by other authors [31], Burnett et al. [14] suggested that animal activity may be more important for the changes in RRT than for other body temperature measurements. Variations in the hormones estrogen and progesterone in estrus change behavior (increasing physical activity), increase the metabolism (they are anabolic), and increase the animal’s temperature. At the same time, increased physical activity (movement) during estrus promotes the release of various hormones (e.g., GH, PRL, ACTH, cortisol, TSH, T3, and T4) that also increase the metabolism and body temperature. Thus, all these physiological changes during estrus cause an increase in RRT.

The increase in ACT after implant removal (hour 0), demonstrated in Figure 6b, can be attributed to the behavioral changes that characterize and precede the primary sign of estrus, such as the acceptance of mounts. The results of Sveberg et al. [32] indicate an increase in the frequency of secondary estrus signs in cattle 6 h before mount acceptance. Secondary signs include licking/sniffing the vulva, chin resting, head raising, flehmen response, and mounting. These behaviors can explain the increase in ACT during the periods evaluated in the present study [33,34].

Minegishi et al. [35] also observed in their study an increase in daily activity and a reduction in rumination time close to estrus in animals from two dairy herds using an accelerometer and rumination system. Dolecheck et al. [1], using multiple automated monitoring technologies in Holstein cows, also found an increase in all activity measures during estrus when compared to the non-estrus period.

The RRT and ACT variables analyzed in the present study exhibited variations attributed to the occurrence of estrus; thus, the use of these variables may assist in estrus detection, and they were included as predictor variables in prediction models tested in the present study. In addition to these variables, the THI was included in the model as a predictor of the occurrence of estrus. In fact, Lewis and Newman [36] suggested the adjustment of body temperature variations to the environmental temperature for estrus prediction. The time of day was also included as a predictor since changes in temperature according to the circadian rhythm were observed, with the body temperature increasing during the day, reaching a peak at the end of the day, and declining during the night. Lopes et al. [37] reported the same pattern of daily oscillation in RRT for Nelore cows as observed in our study and the same magnitude of body temperature (39.3 °C). The same pattern was also described for Angus cows [9], but with lower magnitudes of RRT (38.54 + 0.01 °C). Thus, the ability of these variables together to predict estrus was tested using different prediction models: logistic regression, random forest, and linear discriminant analysis. 

In the present study, the differences among the tested prediction models in the ability to identify the positive predictive value (PPV) might be explained by the variability in the number of records of the response variable among animals, with some animals only having one record of the presence of estrus (Yi = 1) throughout the study period and others having 97 records. Minegishi et al. [35] also observed differences in the PPV between the algorithms studied. According to these authors, this variation may occur when the number of estrus alerts generated by the algorithms varies widely. On the other hand, the specificities, that is, the ability to predict non-estrus events, were high for all models, which can be attributed to the 52 h observation period in the present study. Considering that the period of estrus in Zebu animals is shorter [3,4], most records were the absence of estrus (Yi = 0). Reducing the estrus period [1] may increase the accuracy of the models since the mean estrus duration is less than 24 h [32,34,38].

The accuracy was the only metric similar for the three models, and this finding can be attributed to the fact that accuracy provides the proportion of correct predictions without considering what was classified as positive or negative. Using random forest, linear discriminant analysis, and a neural network, Dolecheck et al. [1] achieved 91% and 100% accuracy of estrus detection, percentages higher than those found in the present study. These authors explained the differences in the performance of the machine-learning techniques by the smaller number of observations and the variety of parameters measured by each technology. 

The random forest model provided the best performance according to the AUC metric, very close to the maximum value (1.0). The accuracy of predicting an event increases when the true-positive rates are high and the false-positive rates are low, resulting in higher AUC values [39], a fact that explains the better performance of the random forest model. According to Zhu et al. [40], AUC values between 0.6 and 0.7 can be classified as not good, between 0.71 and 0.80 as worthless, between 0.81 and 0.90 as good, and between 0.91 and 1.0 as excellent for predicting events. Thus, considering this AUC, the performance of the model using RRT, ACT, the THI, and time of day as predictors of estrus can be classified as good for predicting estrus events.

The differences between the performance of the prediction models can be explained by the ability of the algorithms to learn the complex relationship between the input variables (RRT, ACT, THI, and time of day) and the occurrence of estrus, because these techniques use only a fraction of data and the intersection of input variables to train the computer to achieve an answer [41].

The Dairy Gyr breed is a genetic resource widely used in tropical production systems, especially in crossbreeding with European breeds, with Holstein cattle being the most frequently used animals. This crossing is carried out to improve the productivity of milk production systems under tropical conditions since Zebu breeds exhibit excellent adaptations to these conditions, whereas purebred animals of specialized breeds have serious adaptation problems under the same conditions [42]. However, although the duration of the estrus cycle is similar between taurine and zebuine breeds, the estrus period is shorter in Zebu females [43]. Furthermore, estrus occurs in Dairy Gyr cows mainly between the beginning of the night and early morning hours, a fact that can impair the detection of estrus by the producer [7]. Thus, the RRT and ACT data obtained with bolus sensors, combined with environmental data such as temperature and relative humidity, can be of value for improving the reproductive efficiency of Zebu herds in tropical regions.

## 5. Conclusions

The RRT of the Dairy Gyr animals studied increased significantly after acceptance of the first mount, a time when the heifers were sexually receptive. A significant increase in the activity of the animals was observed after removal of the progesterone implant, possibly due to the behavioral changes that precede the primary sign of estrus. Among the models studied, the random forest model exhibited the best performance in predicting the occurrence of estrus. The results suggest that RRT and ACT data obtained with bolus sensors, combined with environmental data such as temperature, relative humidity, and circadian cycle (time of day), can contribute to the identification of estrus and be of value for improving the reproductive efficiency of Zebu herds in tropical regions.

## Figures and Tables

**Table 1:** Mean and standard deviation of the environmental temperature-humidity index (THI) on the days studied.

Day of Synchronization Protocol	Group			
	1	2	3	4
9 *	77.05 ± 4.12	75.86 ± 3.34	73.94 ± 3.65	72.35 ± 4.32
10	74.69 ± 1.72	73.03 ± 2.79	71.97 ± 3.11	74.50 ± 4.62
11 **	73.98 ± 3.64	68.02 ± 3.59	71.15 ± 2.15	73.24 ± 3.13
12	73.17 ± 3.26	66.44 ± 7.44	71.94 ± 4.33	70.76 ± 3.00

* Day of removal of the progesterone implant; ** day of onset of sexual receptivity (estrus) of the animals and of artificial insemination.

**Table 2:** Means and standard errors for reticulo-rumen temperature (RRT) of Dairy Gyr heifers in Schemes 1 and 2 at 2, 4, 6, 8, and 12 h during the different periods evaluated (day before, estrus, and day after).

Scheme 1 (Hours before First Observed Mount Acceptance)			
Time (Hours)	Period		
	Day before	Estrus	Day after
2	39.29 ^a^ ± 0.05	39.36 ^a^ ± 0.04	39.07 ^b^ ± 0.05
4	39.29 ^a^ ± 0.05	39.38 ^a^ ± 0.04	39.11 ^b^ ± 0.05
6	39.31 ^ab^ ± 0.05	39.38 ^a^ ± 0.04	39.17 ^b^ ± 0.05
8	39.32 ^ab^ ± 0.04	39.37 ^a^ ± 0.03	39.23 ^b^ ± 0.04
12	39.30 ^a^ ± 0.04	39.34 ^a^ ± 0.03	39.25 ^a^ ± 0.04
Overall mean	39.30 ± 0.05	39.37 ± 0.04	39.17 ± 0.05
**Scheme 2 (Hours after First Observed Mount Acceptance)**			
2	39.27 ^b^ ± 0.05	39.45 ^a^ ± 0.04	39.12 ^c^ ± 0.04
4	39.28 ^b^ ± 0.04	39.51 ^a^ ± 0.04	39.12 ^c^ ± 0.04
6	39.31 ^b^ ± 0.04	39.55 ^a^ ± 0.04	39.15 ^c^ ± 0.04
8	39.32 ^b^ ± 0.04	39.57 ^a^ ± 0.04	39.18 ^c^ ± 0.03
12	39.31 ^b^ ± 0.03	39.54 ^a^ ± 0.03	39.21 ^b^ ± 0.03
Overall mean	39.30 ± 0.04	39.52 ± 0.04	39.16 ± 0.04

Means in the same row followed by different superscript letters differ significantly (Tukey test, 5% significance level).

**Table 3:** Means and standard errors for activity of Dairy Gyr heifers at 6, 12, 18, and 24 h after removal of the progesterone implant compared to the same daily hours of the day before removal.

Time (Hours)	Period		Difference
	Day before	Day of Implant Removal	
6	6.67 ^b^ ± 1.03	8.93 ^a^ ± 1.04	2.26
12	6.75 ^b^ ± 1.03	9.06 ^a^ ± 1.04	2.31
18	8.95 ^b^ ± 1.03	11.12 ^a^ ± 1.03	2.17
24	9.40 ^b^ ± 1.03	11.35 ^a^ ± 1.03	1.95

Means in the same row followed by different superscript letters differ significantly (Tukey test, 5% significance level).

**Table 4:** Mean and standard deviation of the performance measures of the logistic regression, random forest and linear discriminant analysis models using reticulo-rumen temperature, activity, time of day, and temperature-humidity index as predictors of estrus.

Performance Measure	Model		
	Logistic Regression	Random Forest	Linear Discriminant Analysis
Sensitivity (%)	0 ± 0	51.69 ± 6.53	0.05 ± 0.11
Specificity (%)	99.79 ± 0.13	93.10 ± 0.74	99.65 ± 0.22
PPV (%)	0 ± 0	56.02 ± 5.32	1.92 ± 3.85
NPV (%)	85.35 ± 1.53	91.83 ± 1.41	85.34 ± 1.54
Accuracy	0.85 ± 0.02	0.87 ± 0.01	0.85 ± 0.02
AUC	0.52 ± 0.04	0.90 ± 0.02	0.64 ± 0.04

The value of each measure is the mean of five repeats of cross-validation. PPV = positive predictive value; NPV = negative predictive value; AUC = area under the ROC curve.

## Data Availability

The data presented in this study are available on request from the corresponding author.
